# Effects of oral liquiritigenin inoculation on gut microbiota and gene expression in intestinal and extraintestinal tissues of mice

**DOI:** 10.3389/frmbi.2024.1380152

**Published:** 2024-09-27

**Authors:** Zhaotaize Suo, Ying Yu, Fangyun Shi, Jijing Tian, Zhihui Hao, Jingren Zhang, Jun Zou

**Affiliations:** ^1^ The High School Affiliated to Renmin University of China, Beijing, China; ^2^ College of Veterinary Medicine, China Agricultural University, Beijing, China; ^3^ Tsinghua-Peking Center for Life Sciences, Tsinghua University, Beijing, China; ^4^ Center for Inflammation, Immunity, and Infection, Institute for Biomedical Sciences, Georgia State University, Atlanta, GA, United States

**Keywords:** phytochemicals, flavonoid, liquiritigenin, gut microbiota, RNA-seq analysis, gene expression, dysbiosis, mucosal immunity

## Abstract

**Introduction:**

Liquiritigenin (LQ), a natural flavonoid found in traditional Chinese medicine and often administered orally, holds potential to affect both the gut and its microbiota, that potentially mediating or influencing its biological and pharmacological effects. However, the effects of LQ on gut microbiota composition and intestinal function remain poorly understood. In this study, we aimed to explore the impact of LQ on gut microbiota and gene expression in both intestinal and extraintestinal tissues.

**Methods:**

We orally inoculated six-week-old SPF C57BL/6 mice with either LQ (a concentration of 4 mg/ml diluted in dimethylsulfoxide, (DMSO)) or DMSO, and administered daily for a duration of 2 weeks. At the end of the experimental period, all mice were euthanized. Fresh fecal samples, as well as samples from the intestine, lung, and liver, were collected for subsequent microbiota analysis, RNA-seq, or histochemical and immunohistochemical (IHC) staining.

**Results:**

Findings show that LQ alters gut microbiota composition, enhancing microbial correlations in the colon but causing some dysbiosis, evidenced by increased pathobionts, decreased beneficial bifidobacteria, and reduced microbiota diversity. Gene expression analysis reveals LQ upregulates mucosal immune response genes and antiinfection genes in both the intestine and lung, with histology confirming increased Paneth cells and antimicrobial peptides in the intestine. Additionally, LQ affects tissue-specific gene expression, triggering hypersensitivity genes in the colon, downregulating metabolic genes in the small intestine, and reducing cell motility and adhesion genes in the lung.

**Discussion:**

These results suggest LQ’s potential to modulate common mucosal immunity but also highlight possible risks of gut dysbiosis and hypersensitivity, particularly in vulnerable individuals. Our study, while informative about the effects of LQ on gut health, lacks direct evidence on whether changes in gut microbiota and gene expression caused by LQ impact inflammatory diseases or are causally linked. Future research should investigate this through fecal microbiota transplantation to explore the causal relationships and LQ’s potential effects on immune responses and disease outcomes in relevant models.

## 1 Introduction

Phytochemicals are a diverse group of bioactive compounds found in plants, which have gained significant attention due to their potential health benefits and therapeutic properties (Ramalingam et al., 2018). Among these phytochemicals, liquiritigenin (LQ), a flavonoid predominantly found in the roots of *Glycyrrhiza glabra* (licorice), has been reported to exhibit various biological activities, such as anti-infection, antioxidant, and anticancer properties (Kim et al., 2008; Liu et al., 2012; Chen et al., 2014; Ji et al., 2021). Many therapeutic formulations containing LQ are administered orally, necessitating their passage through the gastrointestinal tract. This interaction between LQ and the digestive system suggests that LQ may have significant effects on gut microbiota and overall intestinal health (Wang et al., 2015). However, despite LQ’s promising attributes, the full extent and nature of its impact on gut microbiota and intestinal function remain largely unknown (Ramalingam et al., 2018). This highlights the need for further detailed studies to elucidate how LQ influences these crucial aspects of human health, potentially guiding the development of more effective phytochemical-based therapeutics. Empirical research has demonstrated LQ’s capability to inhibit the growth of various bacteria and viruses and to suppress tumor growth, indicating its potential to impact host immune status positively (Wang et al., 2015). The gut microbiota plays a crucial role in sustaining host health by modulating immune responses, metabolism, and other physiological processes (Shen et al., 2013; Belcheva et al., 2014; Wang et al., 2023). A balanced gut microbial community is essential for the proper functioning of the gastrointestinal tract and overall well-being (Selma et al., 2009; Verbeke et al., 2015). However, factors such as diet, drug, and environmental exposure can disrupt this delicate balance, leading to dysbiosis and potentially contributing to the development of various diseases (Belcheva et al., 2014). Therefore, understanding the effects of drug bioactive compounds like LQ on gut microbiota and host immunity is crucial to use this drug properly to maximize its effectiveness, avoid its side effects.

Gene expression is a key factor in determining the functional status of cells and tissues and can be modulated by various external factors, including dietary components (Wang et al., 2021b, 2022). Recent studies have indicated that certain phytochemicals can influence gene expression profiles in different tissues, which may contribute to their health-promoting effects (Wang et al., 2022). Exploring the impact of LQ on gene expression in both intestinal and extraintestinal tissues of mice offers valuable insights into the mechanisms through which it exerts diverse biological activities, spanning anti-infection, antioxidant, and anticancer properties, and its potential role in disease prevention. This study was conducted to investigate the effects of oral inoculation of LQ on gut microbiota and gene expression in intestinal and extraintestinal tissues. Our findings showed that LQ treatment has a dual effect: it enhances gut microbial correlations in the colon but also causes some gut dysbiosis characteristics. Meanwhile, such changes may enhance the mucosal immune response to pathogen infection while triggering hypersensitivity, which serves as a warning for its use in inflammatory diseases. Understanding these mechanisms is integral to harnessing the benefits of LQ treatment effectively while mitigating associated risks and side effects.

## 2 Materials and methods

### 2.1 Animals

Six-week-old specific pathogen-free (SPF) C57BL/6 mice were obtained from Beijing Huafukang Biotechnology Co., Ltd. (Beijing, China). The mice were provided with a pathogen-free diet and access to water *ad libitum* throughout the study. Animal experiments were conducted with approval from the Beijing Administration Committee of Laboratory Animals, and all procedures adhered to the guidelines outlined by the China Agricultural University Institutional Animal Care and Use Committee (AW122022-1-2), following the International Guiding Principles for Biomedical Research Involving Animals.

### 2.2 Chemicals and antibodies

Liquiritigenin (CAS No.: 69097-97-8) and dimethylsulfoxide (CAS No.: 67-68-5) were procured from Sigma-Aldrich (Shanghai, China). Ki67 polyclonal antibody (Cat No. 28074-1-AP, Proteintech Group) and PCNA Polyclonal antibody (Cat No. 10205-2-AP, Proteintech Group) were sourced from M&C Gene Technology Ltd. (Beijing, China).

### 2.3 Liquiritigenin treatment and sample collection

Six-week-old SPF C57BL/6 mice were randomly assigned to two groups (n=8 mice per group): LQ treatment group and the vehicle (dimethylsulfoxide, DMSO) group. Mice in the LQ group received oral inoculation of 20 mg/kg of body weight LQ, a dosage established based on previous studies demonstrating its efficacy in mouse models (Ramalingam et al., 2018). LQ was diluted in DMSO to a concentration of 4 mg/ml and administered daily for a duration of 2 weeks. The vehicle group received an equivalent volume of the vehicle alone for the same duration. Throughout the experiment, the mice were closely monitored for any clinical symptoms or abnormalities. At the end of the experimental period, all mice were euthanized. Fresh fecal samples, as well as samples from the intestine, lung, and liver, were collected for subsequent microbiota analysis, RNA-seq, or histochemical and immunohistochemical (IHC) staining.

### 2.4 Histology and immunohistochemistry

Freshly collected intestinal tissues were fixed in 4% paraformaldehyde (Beyotime), embedded in paraffin, and sectioned. Hematoxylin and eosin staining was performed for histological assessment. For AB-PAS staining, sections were sequentially treated with AB-PAS C, B, and A solutions, rinsed, dehydrated, and sealed. Phloxine B staining involved dewaxing, hydration, staining with hematoxylin and phloxine B solution, rinsing, dehydration, xylene treatment, and sealing with neutral gum. IHC analysis was conducted on 4 μm sections. After de-paraffinization and rehydration, antigen retrieval was performed using Tris EDTA (pH 9.0) via a pressure cooker. Sections were then blocked with 10% goat serum and incubated with primary antibodies against anti-mouse Ki67 (Proteintech) or PCNA (Proteintech) overnight at 4°C or for 2 hours at room temperature. Immunostaining was carried out using a kit from Vector Laboratories following the manufacturer’s protocol. Analysis of the processed tissue sections was performed using an Olympus IX71 microscope and an automated digital slide scanner at Wuhan Servicebio Technology Co., Ltd. (Wuhan, China).

### 2.5 16S rRNA sequencing and microbiota analysis

Fresh individual fecal and small intestinal content samples from 8 mice per group were collected at 2 weeks post LQ or vehicle treatment, blended, and stored at -80°C in sterile 1.5 mL centrifuge tubes. Microbial genomic DNA extraction was performed using the QIAamp DNA Stool Mini Kit by QIAGEN following the manufacturer’s instructions (Li et al., 2003). The V3-V4 hypervariable regions of the 16S rRNA gene were PCR-amplified from the extracted genomic DNA using barcoded fusion primers (338-F: 5’-ACTCCTACGGGAGGCAGCAG-3’ and 806-R: 5’-GGACTACHVGGGTWTCTAAT-3’) (Gong et al., 2022). Sequencing of the amplified products was conducted on the Illumina Miseq PE300 platform by Shanghai Majorbio Bio-pharm Technology (Shanghai, China) (Ni et al., 2022). Sequences not meeting quality standards—such as primer mismatches, lengths shorter than 100 bp, homopolymer runs exceeding 6, and uncertain bases—were excluded. Paired end reads of V3 and V4 that had an overlap of more than 10 bp without mismatches were merged based on their overlapping sections; unassembled reads were removed. The refined sequences underwent taxonomic classification by comparison with Silva v138 databases. A correlation heatmap illustrating the association between cell differentiation-associated differentially expressed genes (DEGs) and bacterial genera was created using the vegan package in R. Additionally, SPSS 19.0 software was employed to conduct a Pearson correlation analysis. Data analysis, including both heatmap generation and correlation analysis, was carried out by Shanghai Majorbio Bio-pharm Technology in China.

#### 2.5.1 RNA extraction and RNA-seq

Total RNA from intestine, lung, or liver tissues was isolated using TRIzol^®^ reagent. RNA quality was assessed using the Agilent 5300 Bioanalyzer and quantified with the ND-2000 (NanoDrop Technologies). RNA purification, reverse transcription, library construction, and sequencing were conducted at Shanghai Majorbio Bio-pharm Biotechnology Co., Ltd. (Shanghai, China) following Illumina’s guidelines (Illumina, San Diego, CA). The RNA-seq library for mice was prepared with the Illumina^®^ Stranded mRNA Prep Ligation Kit from Illumina (San Diego, CA) using 1 μg of total RNA. Initially, mRNA was isolated through polyA selection using oligo(dT) beads. Subsequently, it was fragmented using a specific buffer. Double-stranded cDNA was synthesized with the SuperScript kit (Invitrogen, CA) and random hexamer primers from Illumina. This cDNA underwent end-repair, phosphorylation, and ‘A’ base addition as per Illumina’s protocol. The resulting libraries were size selected for 300 bp cDNA fragments using 2% Low Range Ultra Agarose and amplified for 15 cycles with Phusion DNA polymerase (NEB). Post quantification with Qubit 4.0, the RNA-seq library was sequenced on the NovaSeq 6000 with a read length of 2 × 150 bp. The raw reads underwent quality assessment and trimming using Fastp, followed by alignment to a reference genome through HISAT2 (Kim et al., 2015). StringTie was employed for assembling the mapped reads of each sample using a reference-based approach (Pertea et al., 2015).

### 2.6 Differential expression analysis and functional enrichment

Transcripts per million reads (TPM) was utilized to quantify the expression level of each transcript, employing RSEM for gene abundance estimation. Differential expression analysis was conducted using either DESeq2 or DEGseq. Specifically, genes displaying |log2FC|≧1 and FDR≤ 0.05 (DESeq2) or FDR ≤0.001 (DEGseq) were considered statistically significant. Further analysis involved Gene Ontology (GO) and Kyoto Encyclopedia of Genes and Genomes (KEGG) enrichment assessments to identify DEGs significantly enriched in GO terms and metabolic pathways. Enrichment was determined against the entire transcriptome background, and significance was established at a Bonferroni-corrected P-value ≤0.05. GO analysis was executed using Goatools, while KEGG evaluations were conducted through KOBAS (Xie et al., 2011).

### 2.7 Statistical analysis

The data are presented as the mean ± SEM. Statistical analysis was performed using GraphPad Prism 8.0. T-tests and Mann-Whitney test were utilized to determine the significance of differences between distinct treatments. Statistical significance was denoted as *P < 0.05, and **P < 0.01. Conversely, non-significant differences (P > 0.05) were indicated as ns.

## 3 Results

### 3.1 Liquiritigenin impacts the microbiota composition in both small intestine and colon

**Figure 1:**
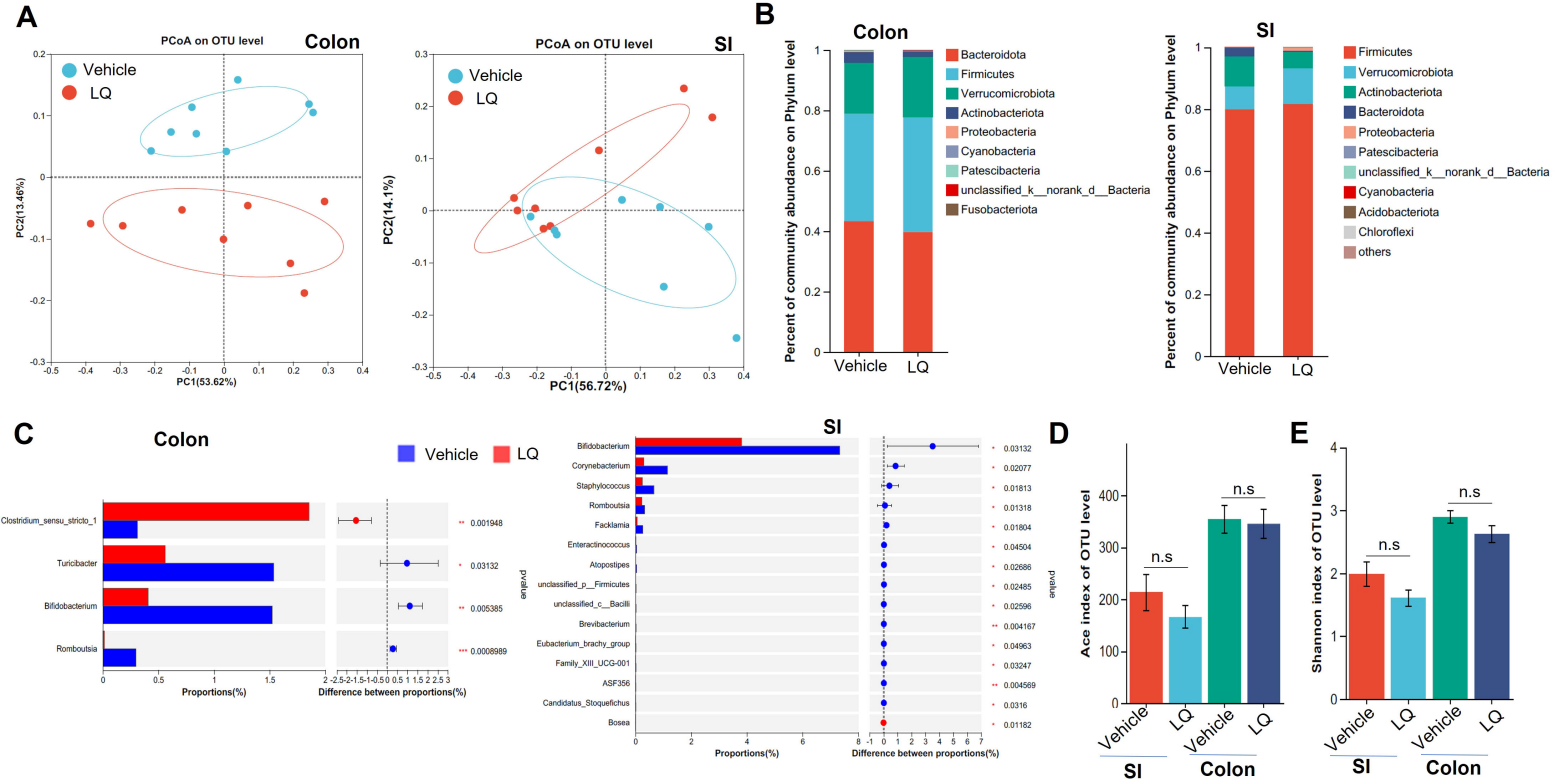
Changes in Fecal Microbiota following Liquiritigenin Treatment. C57BL/6 mice (n=8) received daily oral inoculation of liquiritigenin (LQ) or vehicle (DMSO) for 2 weeks, with fresh fecal samples in colon and small intestine (SI) collected for subsequent microbiota analysis. **(A)** Principal Coordinates Analysis (PCoA) displaying microbiota community clustering. **(B)** Column diagram illustrating community composition at the phylum level. **(C)** Relative abundance comparison of genera exhibiting significant differences between LQ and vehicle groups. D&E. Evaluation of gut microbiota diversity and richness using Ace **(D)** and Shannon indices **(E)**. Data are presented as mean ± SEM. T-tests and Mann-Whitney tests were used to evaluate differences between treatments. Significance is marked as *P < 0.05 and **P < 0.01; ***P < 0.001, non-significant results are noted as ns (P > 0.05).

To investigate the potential of LQ in modulating gut microbiota composition, we administered LQ orally to mice for 2 weeks. Subsequently, we analyzed the gut microbiota communities in colonic feces and small intestine contents using 16S ribosomal RNA (rRNA) gene sequencing. We selected 1,798,769 high-quality sequences, with an average of 56,211 sequences per sample, and identified a total of 16,358 Operational Taxonomic Units (OTUs). The rarefaction curves and the Shannon diversity index based on these OTUs reached plateaus, suggesting that we had captured most of the diversity (**Supplementary Figures S1A, B**). A Venn diagram, based on these OTUs, illustrated the common and specific OTUs in the small intestine and colon, with or without LQ treatment, and suggested that LQ treatment may change the microbiota in both small intestine and colon (**Supplementary Figure S1C**). Furthermore, β-diversity analysis, conducted through Principal Coordinates Analysis (PCoA) and Non-metric multidimensional analysis (NMDS) using the OTU information, highlighted a clear separation in microbiota composition between the small intestine and large intestine without LQ treatment (**Supplementary Figure S1D**), consistent with previous reported (Kastl et al., 2020). Importantly, we also observed varying degrees of impact on the gut microbiota in the small intestine and large intestine after LQ treatment. Specifically, the bacterial structure was distinctly separated between LQ-treated and vehicle-treated groups in colonic feces but showed partial overlap in the small intestine (**Figure 1A**). The noticeable divergence in bacterial composition was prominently visualized at the phylum level using Circos (**Supplementary Figure S2A**). Specially, mice treated with LQ exhibited a trend of increased abundance of Verrucomicrobia and Firmicutes, alongside a decrease in Actinobacteria and Bacteroides in their intestinal microbiota (**Figure 1B**). Notably, Actinobacteria exhibited significant reduction in both the small intestine and colon (**Supplementary Figure S2B**).

Using linear discriminant analysis effect size, we identified various taxa with differing abundances between mice treated with LQ and those receiving the vehicle treatment (**Supplementary Figure S2C**). Specifically, LQ treatment resulted in a significant decrease in the abundance of bacterial genera such as *Bifidobacterium* and *Romboutsia* in both the small intestine and colon (**Figure 1C**; **Supplementary Figure S2C**). Moreover, in the small intestine, we observed a more pronounced decrease in the abundance of bacteria genera, including *Staphylococcus*, *Corynebacterium*, *Facklamia*, and others. Conversely, we observed an increase in the abundance of a few bacterial taxa, including f-Clostridiales in both the small intestine and colon, as well as Bosea, which exhibited an increase limited to the small intestine (**Figure 1C**; **Supplementary Figure S2C**). α-diversity analysis indicated that LQ treatment resulted in a modest, albeit non-significant, reduction in gut microbiota richness and diversity indexes (Shannon, ACE) within both the colon and small intestine (**Figure 1D**).

**Figure 2:**
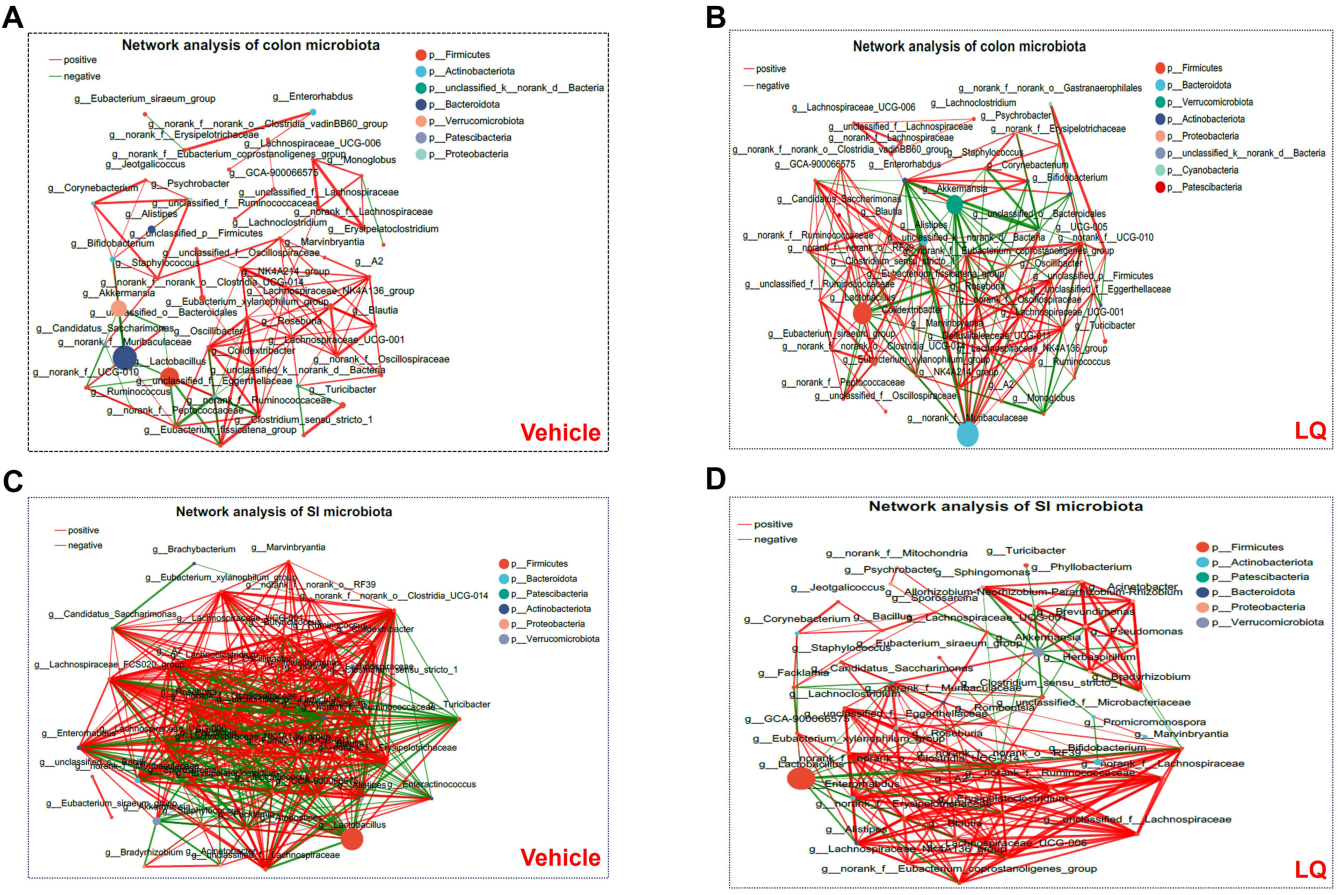
Bacterium-Bacterium Ecological Networks in the Gut of Mice Treated with LQ and Vehicle. Co-occurring bacteria at the genus level are depicted in vehicle-treated **(A, C)** and LQ-treated **(B, D)** groups within the colon **(A, B)** and small intestine (SI, **C**, **D**). Node size and color represent relative abundance and differences in the microbiota. Positive and negative correlations are indicated by red and green lines, respectively. Each group consisted of 8 mice.

Furthermore, we conducted a co-occurrence analysis of gut microbiota in mice to examine the impact of LQ treatment on interspecies interactions. In the colonic microbiota, the vehicle group displayed 95 positive and 23 negative correlations, whereas the LQ group exhibited 142 positive and 64 negative correlations (**Figures 2A, B**). This suggests that LQ treatment enhances the correlation networks within the colon’s microbiota. Notably, we observed that Oscillospiraceae in the Firmicutes phylum played a pivotal role in interspecies interactions within the vehicle group (**Figure 2A**). However, following LQ treatment, the Muribaculaceae in the Bacteroidetes phylum emerged as robust correlations and assuming a central position in the colonic microbiota’s ecological network (**Figure 2B**). Moreover, within the Actinobacteria phylum, which exhibited a significant reduction after LQ treatment, the genus *Bifidobacterium* showed significant positive correlations with *Corynebacterium* and negative correlations with Muribaculaceae in the colon of both vehicle and LQ-treated mice.

Comparing the colonic microbiota, the small intestinal microbiota in vehicle-treated group displayed a stronger correlation network (351 positive and 131 negative correlations). Although LQ treatment did not significantly alter the total number of correlations (334 positive and 120 negative correlations post-treatment), it did change the overall clustering pattern of microbiota in small intestine (**Figures 2C, D**). For instance, in the vehicle group, the genera g:norank, g:A2, g:Lachnospiraceae_FCS020_group, and g:family_XIII_UCG-001 showed the highest correlations with other bacteria, while in the LQ-treated group, g:Eubacterium and g:Enterorhabdus exhibited the highest correlations (**Figures 2C, D**). These shifts in the gut microbiota’s ecological network indicate a significant alteration in interspecies interactions due to LQ treatment.

### 3.2 Liquiritigenin differently impacts the gene expression in colon and small intestine

**Figure 3:**
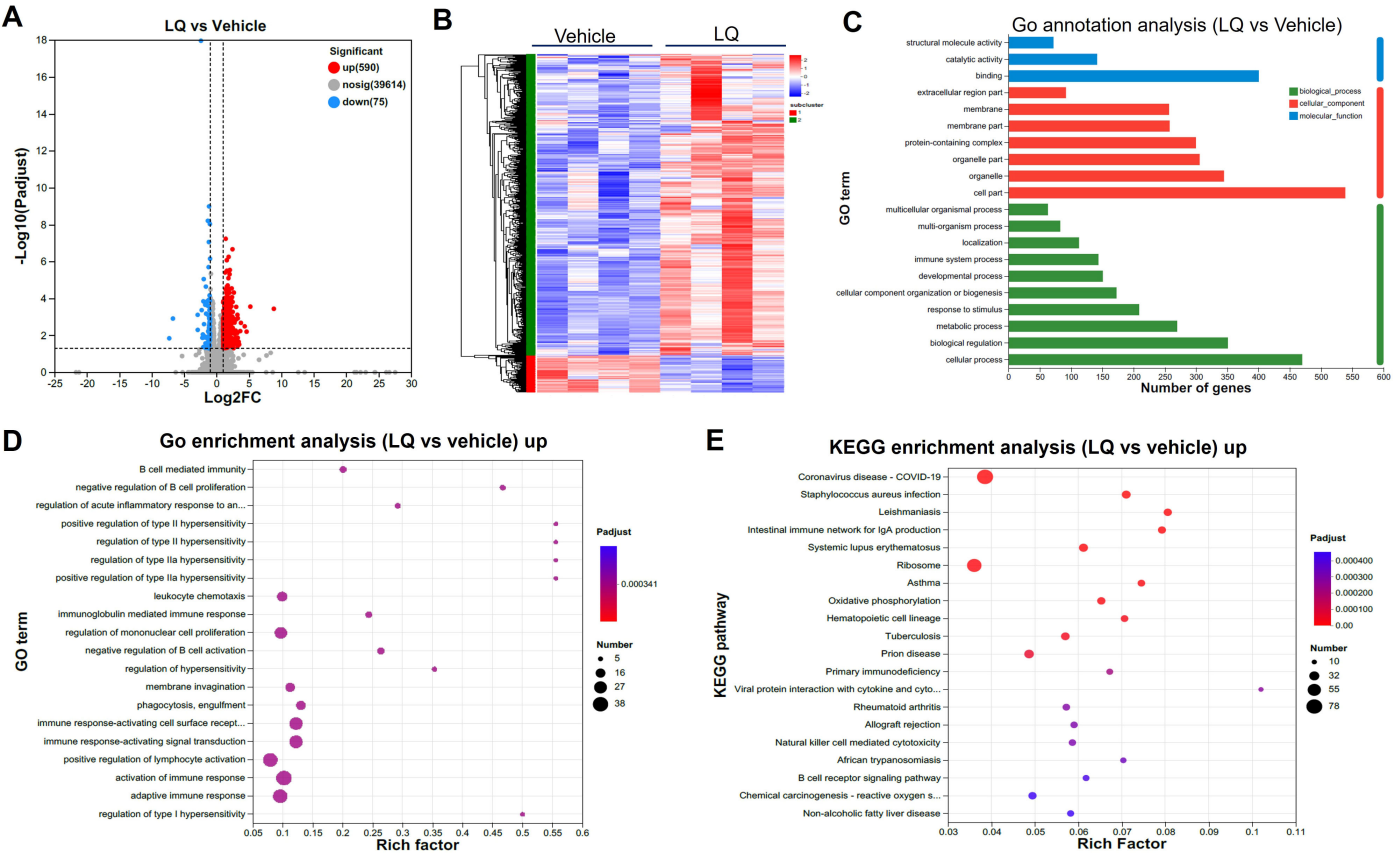
Intestinal Transcriptomic Analysis of Colon in Mice Treated with Liquiritigenin. **(A)** Volcano plots depicting DEGs (Differentially Expressed Genes) between the LQ and vehicle treatment groups (n=4 per group). **(B)** Heatmap displaying DEGs between the LQ and vehicle treatment groups. **(C, D)** GO annotations **(C)** and enrichment **(D)** analysis of DEGs in the LQ versus vehicle treatment groups. **(E)** KEGG enrichment analysis of upregulated genes in the LQ group compared to the vehicle group.

The modification of gut microbiota composition induced by LQ treatment may potentially influence intestinal function. To explore this possibility, we conducted an analysis of gene expression profiles in the colon and small intestine using RNA-seq techniques, aiming to investigate alterations in intestinal function after LQ treatment. The transcriptome analysis of the colon revealed a total of 665 differentially expressed genes (DEGs) in LQ-treated mice when compared to mice treated with the vehicle (590 upregulated and 75 downregulated, **Figure 3A**). To visually depict this difference, we created a heatmap that illustrates the expression patterns of the identified DEGs in both vehicle and LQ treated mice. Notably, cluster analysis of the heatmap provided further evidence of substantial differences in gene expression between the two groups (**Figure 3B**). Moreover, to unveil the biological processes, molecular functions, and cellular compartments associated with the identified DEGs, we conducted Gene Ontology (GO) analysis (**Figure 3C**). This analysis provided insights into the functional implications of these gene alterations. Notably, our observations suggest that LQ treatment may have a substantial influence on increasing immune cells function and inflammatory response in the colon. This is exemplified by the significant enrichment of DEGs associated with activation of immune response, regulation of mononuclear cells proliferation, positive regulation of type II hypersensitivity, immune response-activating cell surface receptor and signal transduction among others (**Figure 3D**). Conversely, downregulation was observed in categories related to cell differentiation and tissue development (**Supplementary Figure S3A**). Along with enhanced immune function by GO enrichment analysis, KEGG analysis indicated that LQ treatment might bolster resistance against pathogen infections. This was evidenced by the enrichment of protective genes associated with infectious diseases such as coronavirus disease, *Staphylococcus aureus* infection, tuberculosis, among others (**Figure 3E**). This enrichment potentially elucidates the anti-infectious disease and immune-regulatory functions attributed to LQ.

**Figure 4:**
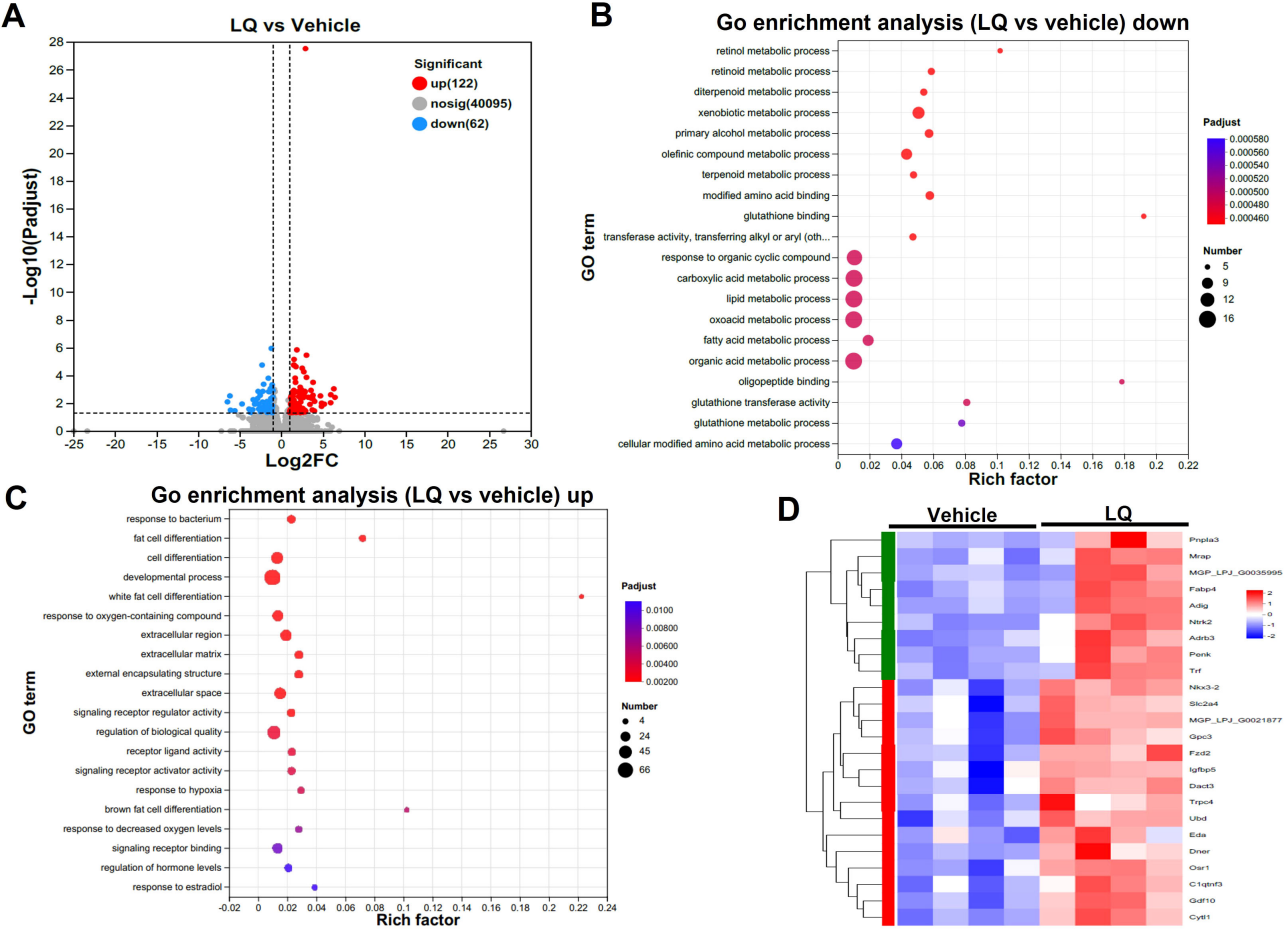
Intestinal Transcriptomic Analysis of Small Intestine in Liquiritigenin-Treated Mice. **(A)** Volcano plots displaying DEGs (Differentially Expressed Genes) between the LQ and vehicle treatment groups (n=4 per group). **(B, C)** GO enrichment analysis of downregulated **(B)** and upregulated **(C)** DEGs in the LQ versus vehicle treatment groups. **(D)** Heatmap representation of DEGs associated with cell differentiation in the LQ versus vehicle treatment groups.

In contrast to its impact on the colon, LQ treatment had a milder effect on gene expression in the small intestine, leading to the upregulation of 122 genes and the downregulation of 62 genes (**Figure 4A**). Similar to the colon, the most enriched terms in molecular function, cellular component, and biological process of small intestine were binding, cell part, and cellular process, respectively (**Supplementary Figure S3B**). Furthermore, cluster analysis of the heatmap (**Supplementary Figure S3C**), which identified differentially expressed genes (DEGs) between vehicle-treated and LQ-treated mice, highlighted a distinct functional landscape in the small intestine due to LQ treatment (**Figure 4**). These gene alterations were associated with various biological processes. Notably, there was enrichment of downregulated genes observed in nutrient metabolic processes such as lipid and fatty acid process, primarily occurring in the small intestine (**Figure 4B**). Conversely, an upregulation was noted in genes enriched for responses to bacterial stimuli and hypoxia, developmental processes, and the regulation of hormone levels (**Figure 4C**). Particularly, there was a notable enrichment of genes associated with cell differentiation (**Figure 4D**).

### 3.3 Liquiritigenin increases the differentiation of Paneth cells in small intestine, that correlated with alteration in the abundance of several bacterial species

**Figure 5:**
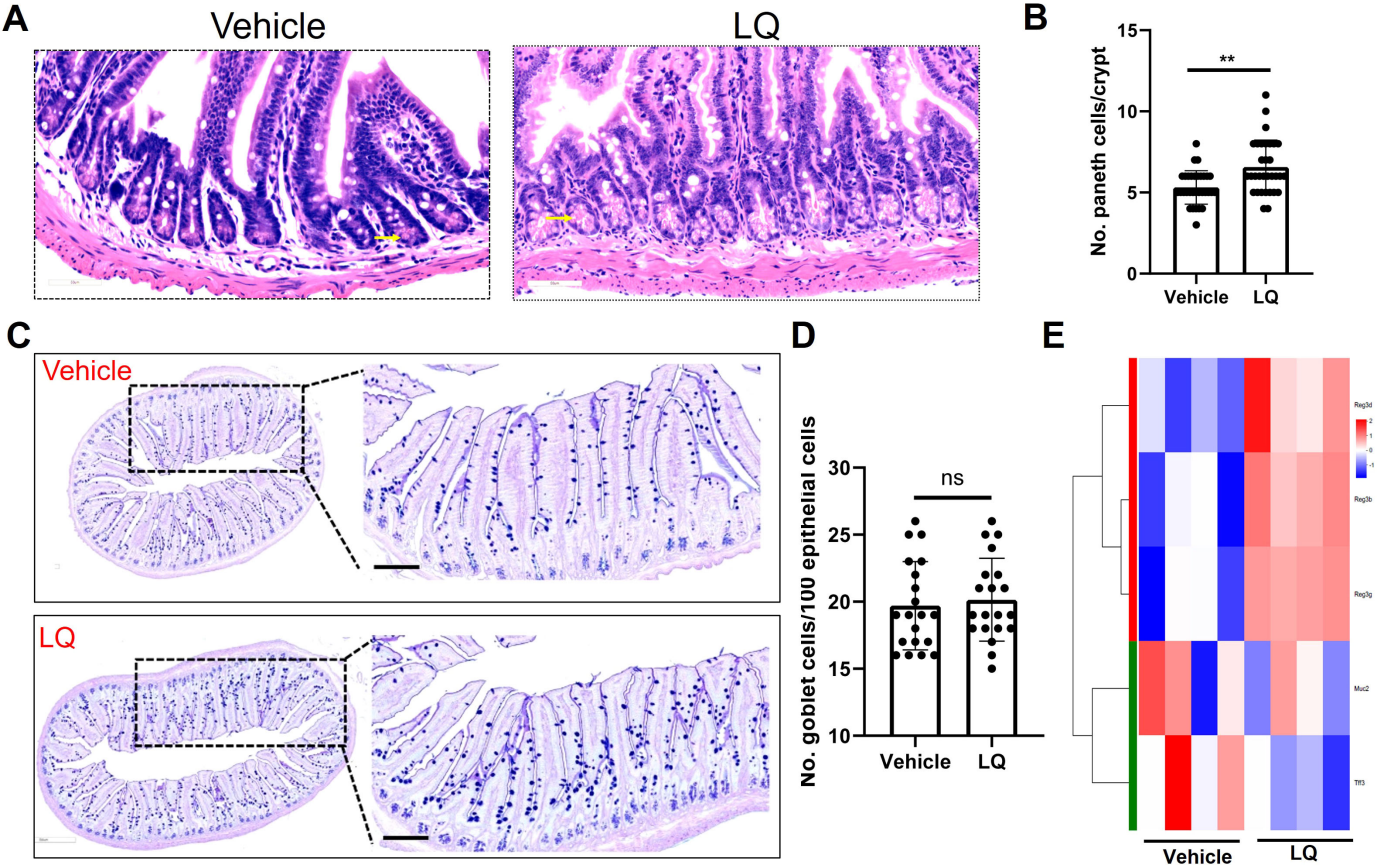
Impact of Liquiritigenin on Paneth Cell Differentiation in the Small Intestine. **(A)** The small intestine (n=4 per group) sections were stained using Hematoxylin and Eosin (HE), and Paneth cells were identified and indicated by arrows. **(B)** Quantification of Paneth cells in randomly selected crypts. **(C)** Visualization and analysis of goblet cells via AB-PAS staining. **(D)** Quantification of the Paneth cell count per crypt. **(E)** Heatmap representation of DEGs linked to Paneth cells and goblet cells in the LQ versus vehicle treatment groups. T-tests was used to evaluate differences between treatments. ** means p < 0.01, ns means p > 0.05.

The RNA-seq analysis indicated the potential of LQ treatment to augment cell differentiation in the small intestine (**Figure 4D**). Consequently, we proceeded to assess the fully differentiated cell population, encompassing Paneth cells and goblet cells, within this intestinal region. The results demonstrated a significant elevation in Paneth cell numbers in LQ-treated mice compared to the vehicle group within the small intestine (**Figures 5A, B**). Additionally, AB-PAS staining analysis of goblet cells revealed that LQ treatment did not increase goblet cells in the small intestine (**Figures 5C, D**). The significantly increasing of Paneth cells differentiation was further supported by significantly increasing of Paneth cells related genes as indicated by RNA-seq data (**Figure 5E**). Conversely, genes associated with goblet cells, including Muc2 and Tff3, did not exhibit an increase (**Figure 5E**). Furthermore, we noted that the upregulation of cell differentiation genes was associated with alterations in several bacterial species in the small intestine, notably a reduction in bifidobacteria due to LQ treatment (**Supplementary Figure S4A**). Additionally, we evaluated the effect of LQ treatment on intestinal stem cell proliferation using Ki67 staining. Our results indicated a moderate, albeit statistically non-significant, increase in the proliferation of intestinal cells (**Supplementary Figure S4B**). Moreover, analysis of the overall intestinal structure showed that LQ treatment did not cause any signs of intestinal epithelial damage or disease (**Supplementary Figure S5**).

### 3.4 The influence of liquiritigenin treatment on gene expression in the liver and lung

**Figure 6:**
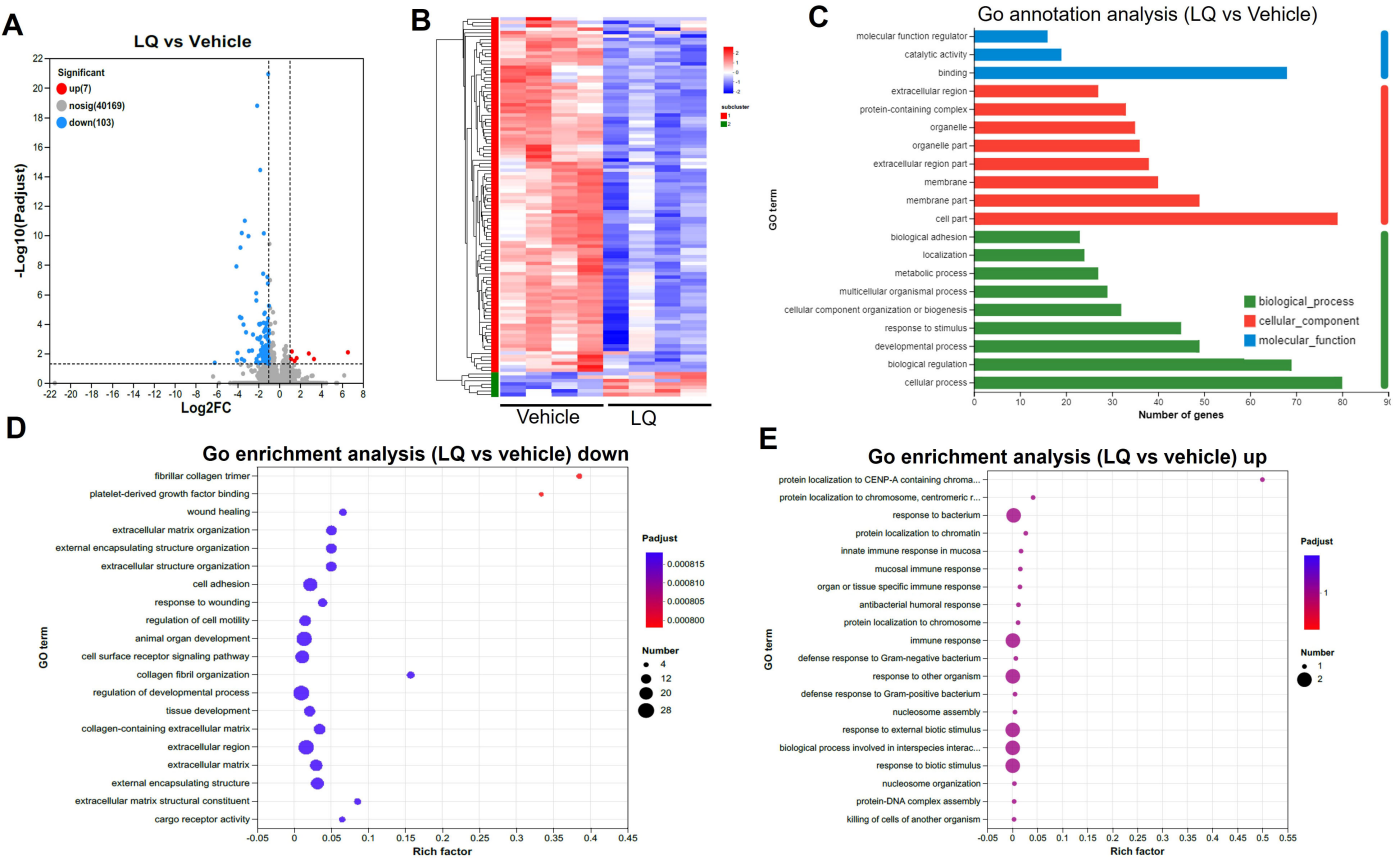
Transcriptomic Analysis of Lung in Liquiritigenin-Treated Mice. **(A)** Volcano plots depict the differential expression of genes (DEGs) between the LQ and vehicle treatment groups (n=4 per group) for lung tissue. **(B)** Heatmap illustrating the expression pattern of DEGs between the LQ and vehicle treatment groups in lung tissue. **(C)** Gene Ontology (GO) annotations analysis of the DEGs in the LQ versus vehicle treatment groups in lung tissue. D&E. Go enrichment analysis displaying downregulated **(D)** and upregulated **(E)** genes in the LQ group compared to the vehicle group in lung tissue.

To explore the impact of LQ treatment on extraintestinal tissues, we conducted gene expression analysis via RNA-seq on both the liver and lung. Interestingly, the gene expression in the liver showed minimal change, with only 2 upregulated and 4 downregulated genes after LQ treatment (**Supplementary Figure S6A**). These differentially expressed genes exhibited a scattered distribution across various pathways based on KEGG analysis (**Supplementary Figure S6B**). In contrast, the analysis of gene expression in the lungs of LQ-treated mice revealed 110 differentially expressed genes, comprising 103 downregulated and 7 upregulated genes (**Figure 6A**). A detailed display of these differentially expressed genes was comprehensively depicted in a heatmap (**Figure 6B**). Subsequently GO enrichment analysis was conducted to delineate the biological functions associated with all the DEGs (**Figure 6C**). The analysis revealed that the most prominent alteration in enrichment degree among these DEGs was observed in the fibrillar collagen trimer category, followed by platelet-derived growth factor binding, as indicated by the Rich factor. The GO enrichment analysis of DEGs revealed notable downregulation in categories associated with cell motility, adhesion, and developmental regulation (**Figure 6D**). Conversely, there was significant upregulation in immune response categories, particularly mucosal and antibacterial humoral responses, among others (**Figure 6E**).

The data presented above demonstrate that LQ treatment alters gut microbiota, enhancing intestinal mucosal immune responses and anti-infection potential. Additionally, LQ treatment extends these effects to the lungs, impacting mucosal immune responses there as well. This suggests that LQ treatment induces a ‘common mucosal response’ across both the intestine and lungs. Moreover, LQ treatment elicited tissue-specific impacts, as evidenced by the enrichment of differentially expressed genes (DEGs) associated with processes such as cell differentiation, structure development, and metabolic activities in the intestine, while in the lung, DEGs were linked to cell motility, cell adhesion, and structural organization. Our analysis further reveals that many of these shared DEGs are upregulated in the intestine but downregulated in the lung (**Supplementary Figures S6C, D**).

## 4 Discussion

Several drugs containing LQ have been proven effective in treating or preventing obesity, infections, and cancer through oral administration (Feldman et al., 2011; Ramalingam et al., 2018; Wang et al., 2021a). In our current study, by utilizing a mouse model, our RNA-seq analysis revealed that LQ treatment led to the upregulation of immune response genes in both the intestine and lungs, potentially mediating its anti-infection properties. Simultaneously, it resulted in the downregulation of genes related to nutrient metabolic processes in the small intestine and cell motility and adhesion in the lungs. These changes may contribute to the prevention of obesity and lung cancer. Interestingly, LQ treatment induced alterations in gut microbiota and enhanced correlation networks within the colon’s microbiota, potentially mediating or influencing its biological and pharmacological effects. However, attention is warranted due to the observed induction of certain aspects of gut dysbiosis by LQ.

In the current study, an intriguing discovery is the alteration of gut microbiota induced by LQ treatment, particularly a notable increase in the “so-called” pathobiont *Clostridium* sensu stricto 1 (Fan et al., 2017) in both the small intestine and colon. Accumulated evidence suggests that certain commensal bacterial strains, termed pathobionts, can exhibit beneficial effects on the host (Jochum and Stecher, 2020). For example, segmented filamentous bacteria did not cause intestinal damage; instead, they enhanced immune defenses and provided protection against certain pathogen infections in immunocompetent mice (Shi et al., 2019). Similarly, *Mucispirillum schaedleri*, identified as another pathobiont, showed no harmful effects in healthy mice while conferring protection against *Salmonella*-induced colitis in these hosts (Herp et al., 2019). Our current study demonstrated that increased immune cell function and inflammatory response, coupled with the absence of epithelial damage or colitis following LQ treatment, as indicated by the enrichment of its related DEGs and histology analysis, associated with the elevation of *Clostridium* sensu stricto 1 in healthy mice, further supporting this notion. However, it should be noted that these pathobionts were found to induce intestinal damage or colitis in severely immune-deficient host, suggesting caution in the use of LQ in immunocompromised patients.

Another intriguing finding is that LQ treatment significantly decreases the abundance of several species, including *Bifidobacterium* spp., in both the small intestine and colon. Bifidobacteria, belonging to the Actinobacteria phylum, are normal inhabitants in the host intestine (O’Callaghan and van Sinderen, 2016). The presence of bifidobacteria in humans has been associated with improvements in overall health conditions, including enhanced immunity, reduced intestinal infections, lower cholesterol, and anti-aging effects, among others (Hidalgo-Cantabrana et al., 2017; Zhao et al., 2018; Chen et al., 2021). Our short-term LQ treatment, which resulted in a reduction of bifidobacteria, did not appear to have immediate side effects. However, considering the potential implications of long-term LQ consumption, it may be advisable to supplement it with probiotic bifidobacteria or prebiotics like inulin, known to specifically promote the growth of bifidobacteria (Zou et al., 2018, 2023). This supplementation could potentially enhance the positive effects of LQ or mitigate any undesirable side effects associated with the reduction of bifidobacteria.

Growing evidence indicates that gut microbiota not only influences mucosal immune responses in the intestine but also has an impact on remote organs, such as the lungs (Keely et al., 2012; Belkaid and Harrison, 2017). Various mechanisms may underlie the influence of gut microbiota on lung immunity (Rastogi et al., 2022). First, the gut microbes and their metabolites may traverse from intestinal sites to stimulate mucosal immune responses in the lungs (Trompette et al., 2014; Anand and Mande, 2018) Second, effector immune cells induced by gut microbiota in the intestine can migrate to the lungs through the lymphatic system, thereby influencing mucosal immune responses in this distant organ, called a common mucosal immunity (Anand and Mande, 2018). Our study demonstrated that LQ treatment led to the upregulation of genes associated with boosting immune response and anti-bacterial infection in the lung rather than the liver, suggested that LQ treatment may induce a common gut-lung mucosal immunity. However, the precise role of gut microbiota in the regulation of lung mucosal immunity induced by LQ treatment remains unclear. This aspect is particularly relevant considering the widespread use of licorice, which contains liquiritigenin as its primary active component, in Traditional Chinese Medicine for the relief of persistent long-term coughs. Therefore, it is crucial to explore the potential mechanisms through which LQ treatment influences lung immunity, particularly in the context of its potential to alleviate persistent coughs, marking an important area for future research.

It is well documented that flavonoids can interact with gut microbiota in a bidirectional manner (Adamczak et al., 2019; Xiong et al., 2023). They have antibacterial properties that can inhibit the growth of certain bacteria while also serving as metabolic substrates that promote the growth of others (Parkar et al., 2013; Farhadi et al., 2019). In our study, we found that liquiritigenin, a plant-derived flavonoid, significantly altered the gut microbiota composition in mice following oral administration. This alteration led to a marked decrease in several species, including Bifidobacterium, in both the small intestine and colon, with the reduction being particularly notable in the small intestine, indicative of its antibacterial activity. Conversely, the abundance of a few bacterial species, notably those in the Clostridiaceae family, increased in both the small intestine and colon after liquiritigenin treatment. This suggests that some Clostridium isolates, which have been shown to degrade flavonoids (Schoefer et al., 2003), might metabolize liquiritigenin, potentially facilitating their growth. This interaction warrants further investigation in future studies to better understand the metabolic relationships between liquiritigenin and specific gut bacteria.

Several flavonoids are known for their beneficial effects, including anti-inflammatory, antioxidant, anti-infection, anti-aging, and anticancer properties (Kurnia et al., 2019; Kopustinskiene et al., 2020). Consistent with these effects, our study found that oral administration of liquiritigenin significantly enhanced the immune response in the colon, evident through increased complement activation, phagocytosis, and B-cell-mediated immunity. These enhancements may help the host fend off bacterial and viral infections and potentially exhibit anticancer activities. However, our study does present limitations that warrant consideration. Firstly, our findings are derived from a single, two-week treatment period with data collected at only one time point, and it is uncertain how these effects might evolve over a longer duration, especially concerning the gut microbiota. Secondly, the study was conducted using normal mice; additional research is necessary to determine if similar outcomes would be observed in disease models or human subjects. Key gaps include exploring the causal relationship between changes in gut microbiota and gene expression induced by LQ treatment and assessing whether these modifications could influence inflammatory-related diseases, such as inflammatory bowel disease and diabetes. To address these limitations and gaps, future research should employ techniques like fecal microbiota transplantation to establish whether changes in gut microbiota induced by LQ have a direct role in mediating its biological effects, including enhancing host immune responses and its potential anti-infection and anticancer properties in disease-specific mouse models with prolonged LQ treatment.

## Data Availability

The datasets presented in this study can be found in online repositories. The names of the repository/repositories and accession number(s) can be found below: PRJNA1082879 and PRJNA1080063.

## Supplementary material

Supplementary Figure S1 Alterations in Fecal Microbiota upon Liquiritigenin Treatment. **(A, B)** Rarefaction and Shannon curves depicting operational taxonomic unit (OTU) diversity and richness. **(C)** Venn diagram illustrating core OTUs shared among LQ and vehicle-treated groups in the small intestine (SI) and colon. **(D)** Principal Coordinates Analysis (PCoA) and Non-metric Multidimensional Scaling (NMDS) plots exhibiting microbiota community clustering in the small intestine and colon within the vehicle-treated groups.

Supplementary Figure S2 Impact of Liquiritigenin Treatment on Fecal Microbiota. **(A)** Circos visualization depicting microbial community distribution at the phylum level. **(B)** Relative abundance comparison of phyla showing significant differences between LQ and vehicle groups. **(C, D)** Bacterial taxa displaying differential enrichment in colon and small intestine (SI) between LQ and vehicle treatments, identified using the effect size (LEfSe) algorithm.

Supplementary Figure S3 Effect of Liquiritigenin Treatment on Gene Expression in the Intestine. **(A)** GO enrichment analysis of downregulated DEGs in the colon of the LQ versus vehicle treatment groups. **(B)** GO annotations analysis of DEGs in the small intestine of the LQ versus vehicle treatment groups. **(C)** Heatmap depicting the expression pattern of DEGs in the small intestine between the LQ and vehicle treatment groups.

Supplementary Figure S4 Correlation heatmap depicting the association between cell differentiation-associated DEGs and bacterial genera. **(A)** Correlation heatmap depicting the association between cell differentiation associated DEGs and bacterial genera. B&C. Cell proliferation in the colon and small intestine was assessed using Ki-67 staining **(B)**, and the density of Ki-67 positive cells was quantified **(C)**.

Supplementary Figure S5 Impact of Liquiritigenin Treatment on the Overall Intestinal Structure. **(A, B)** The overall intestinal structure of the small intestine **(A)** and colon **(B)** was assessed using hematoxylin and eosin (H&E) staining, and villus length and crypt length were quantified.

Supplementary Figure S6 Impact of Liquiritigenin Treatment on Gene Expression in Extraintestinal Tissues. **(A)** Volcano plots illustrating the differential expression of genes (DEGs) between the LQ and vehicle treated groups in the liver. **(B)** Kyoto Encyclopedia of Genes and Genomes (KEGG) enrichment analysis of DEGs in the LQ group compared to the vehicle group in the liver. **(C)** Heatmap illustrating the expression pattern of tissue-shared differentially expressed genes (DEGs) in the small intestine vs lung. **(D)** Heatmap illustrating the expression pattern of tissue-shared DEGs in the colon vs lung.
