# Supplementary figures and images for: Effects of oral liquiritigenin inoculation on gut microbiota and gene expression in intestinal and extraintestinal tissues of mice
Source: Front Microbiomes. 2024 Sep 27;3:1380152. doi: 10.3389/frmbi.2024.1380152 (PMC12993528; doi:10.3389/frmbi.2024.1380152)

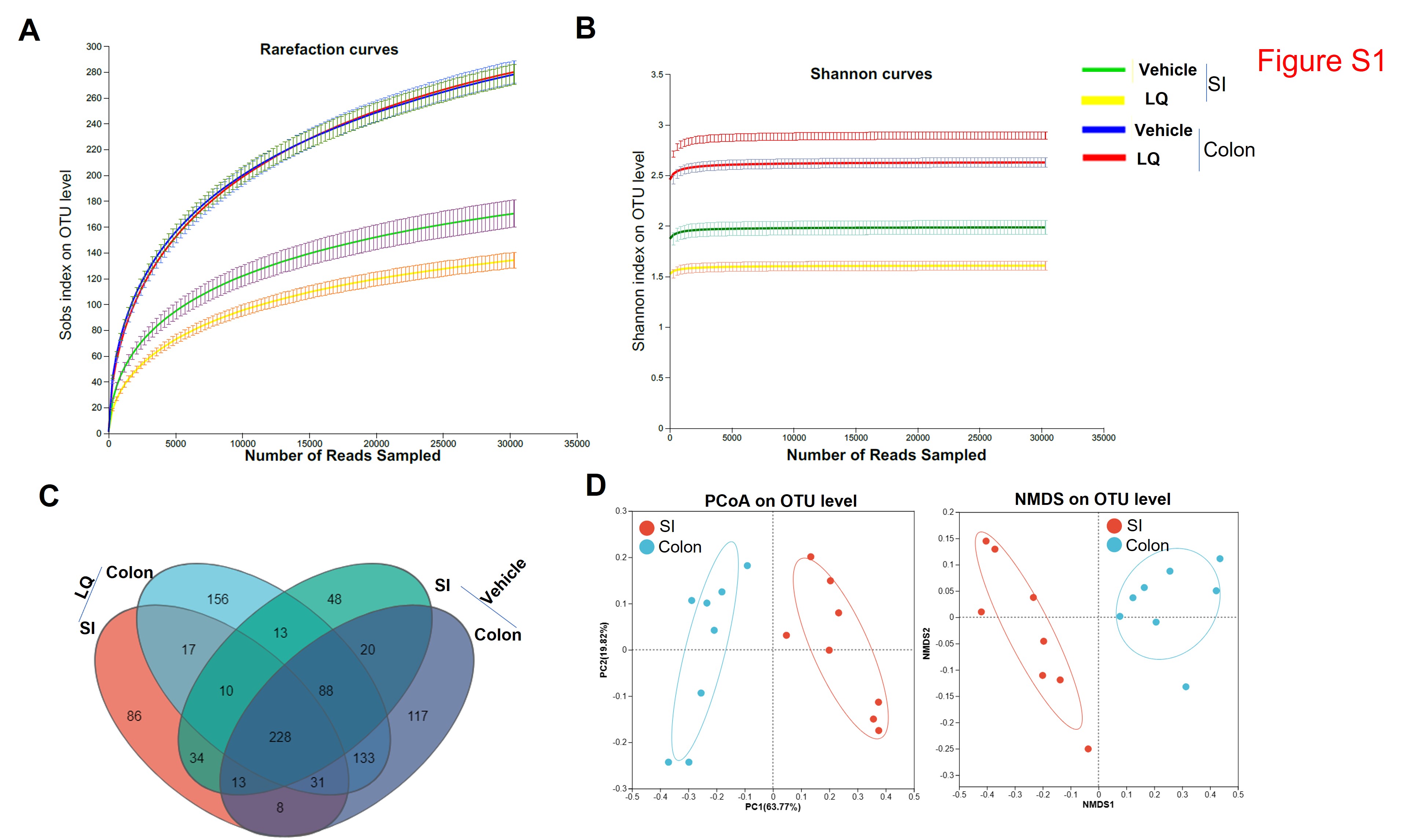

Supplement: Supplementary Figure S1 — Alterations in Fecal Microbiota upon Liquiritigenin Treatment. (A, B) Rarefaction and Shannon curves depicting operational taxonomic unit (OTU) diversity and richness. (C) Venn diagram illustrating core OTUs shared among LQ and vehicle-treated groups in the small intestine (SI) and colon. (D) Principal Coordinates Analysis (PCoA) and Non-metric Multidimensional Scaling (NMDS) plots exhibiting microbiota community clustering in the small intestine and colon within the vehicle-treated groups. [file Image1.jpeg]

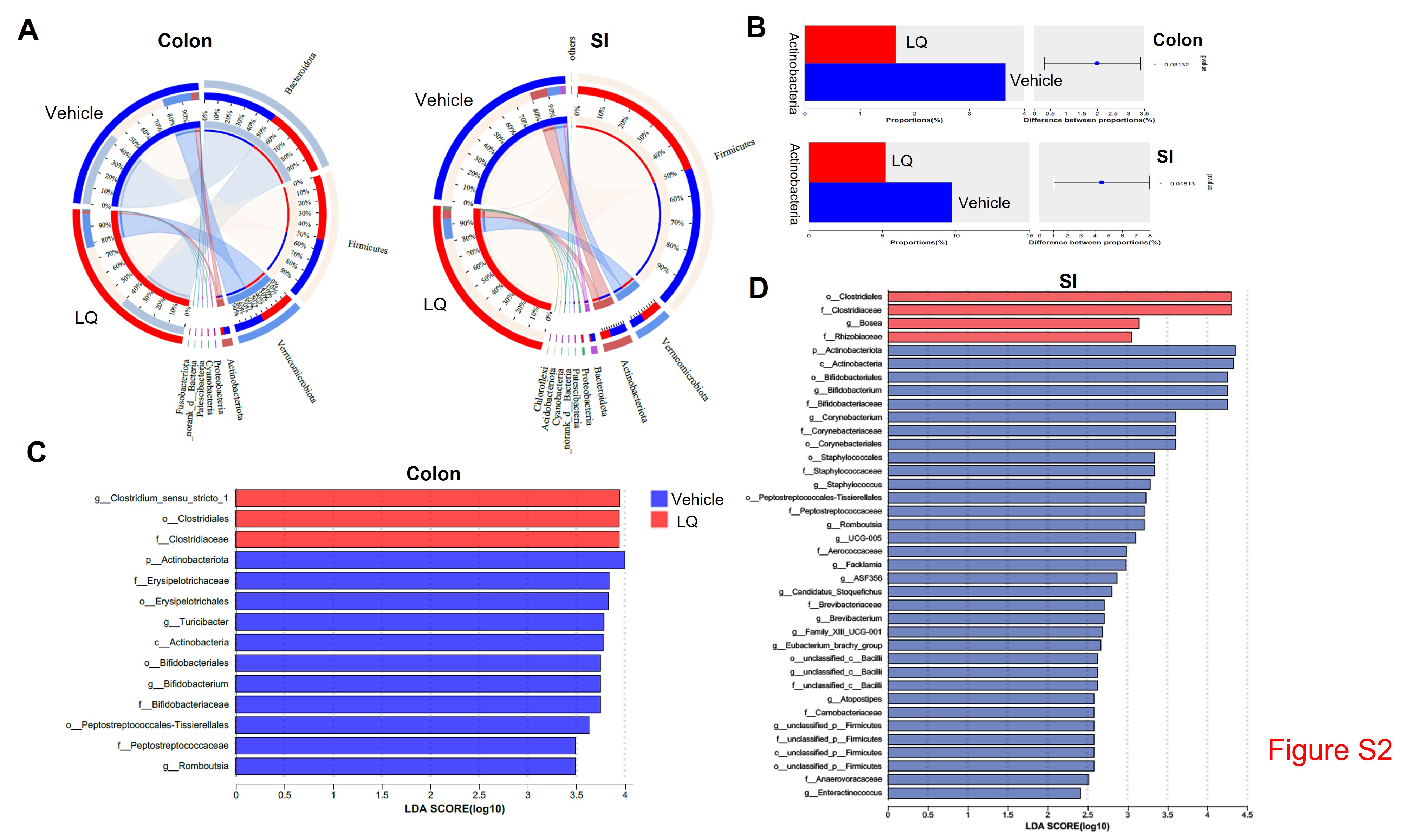

Supplement: Supplementary Figure S2 — Impact of Liquiritigenin Treatment on Fecal Microbiota. (A) Circos visualization depicting microbial community distribution at the phylum level. (B) Relative abundance comparison of phyla showing significant differences between LQ and vehicle groups. (C, D) Bacterial taxa displaying differential enrichment in colon and small intestine (SI) between LQ and vehicle treatments, identified using the effect size (LEfSe) algorithm. [file Image2.jpeg]

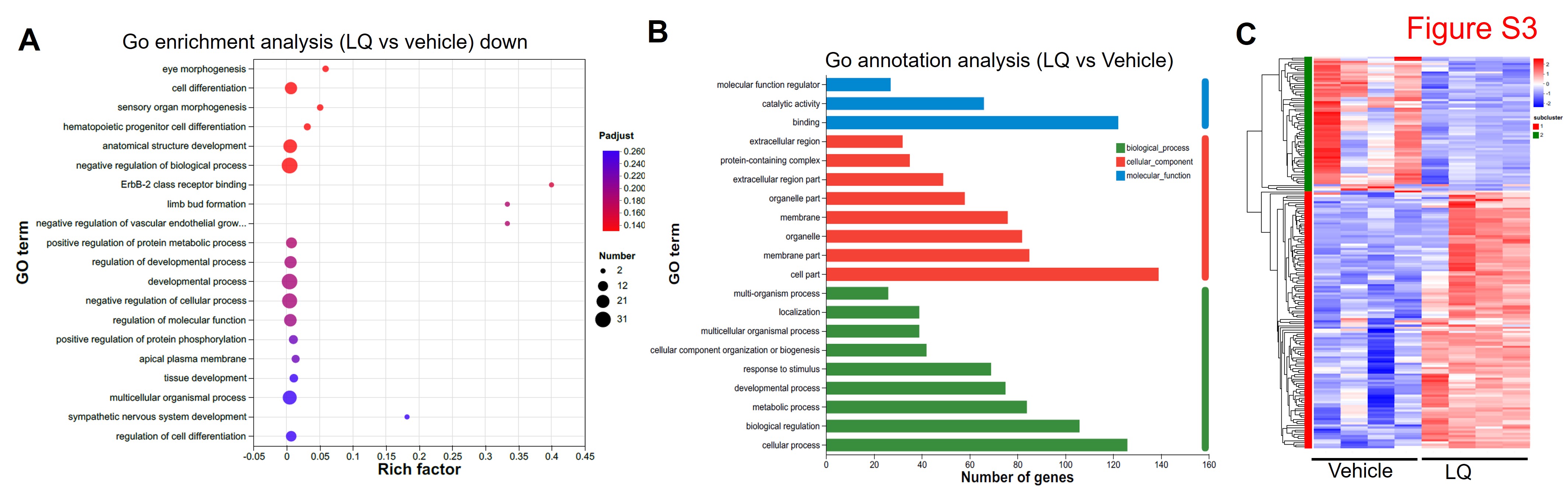

Supplement: Supplementary Figure S3 — Effect of Liquiritigenin Treatment on Gene Expression in the Intestine. (A) GO enrichment analysis of downregulated DEGs in the colon of the LQ versus vehicle treatment groups. (B) GO annotations analysis of DEGs in the small intestine of the LQ versus vehicle treatment groups. (C) Heatmap depicting the expression pattern of DEGs in the small intestine between the LQ and vehicle treatment groups. [file Image3.jpeg]

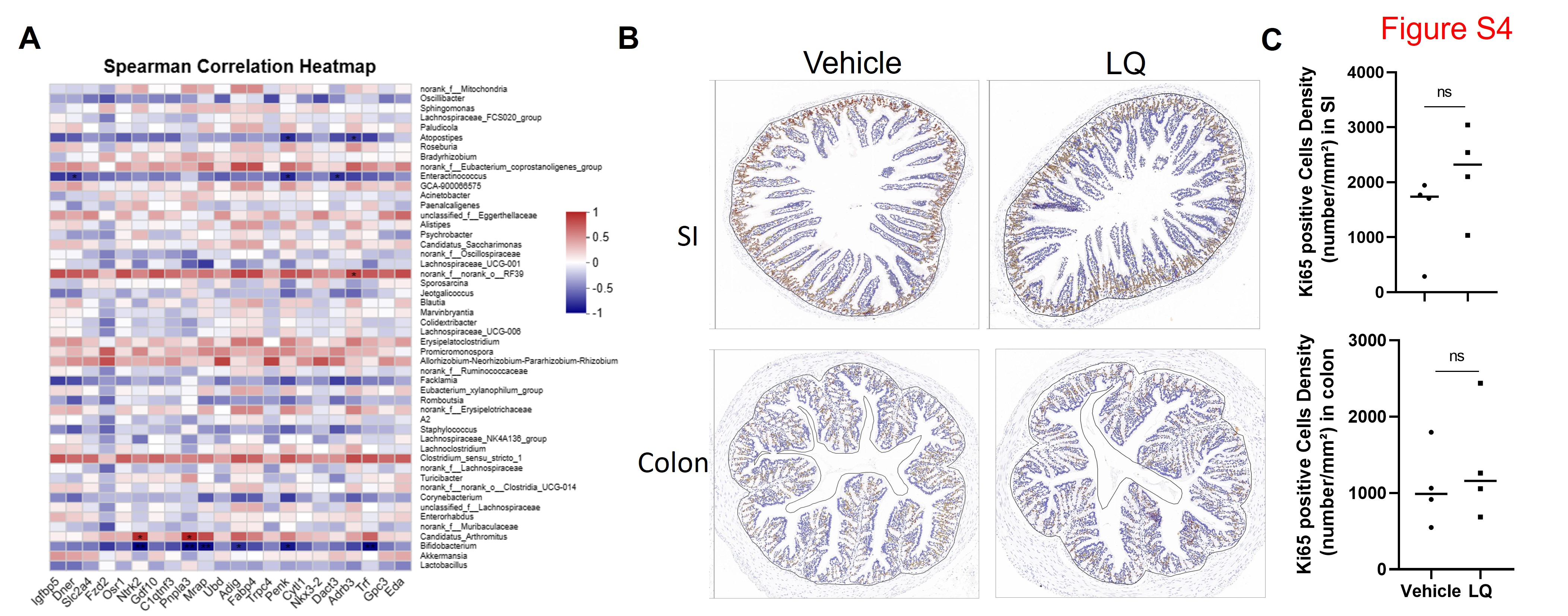

Supplement: Supplementary Figure S4 — Correlation heatmap depicting the association between cell differentiation-associated DEGs and bacterial genera. (A) Correlation heatmap depicting the association between cell differentiation associated DEGs and bacterial genera. B&C. Cell proliferation in the colon and small intestine was assessed using Ki-67 staining (B), and the density of Ki-67 positive cells was quantified (C). [file Image4.jpeg]

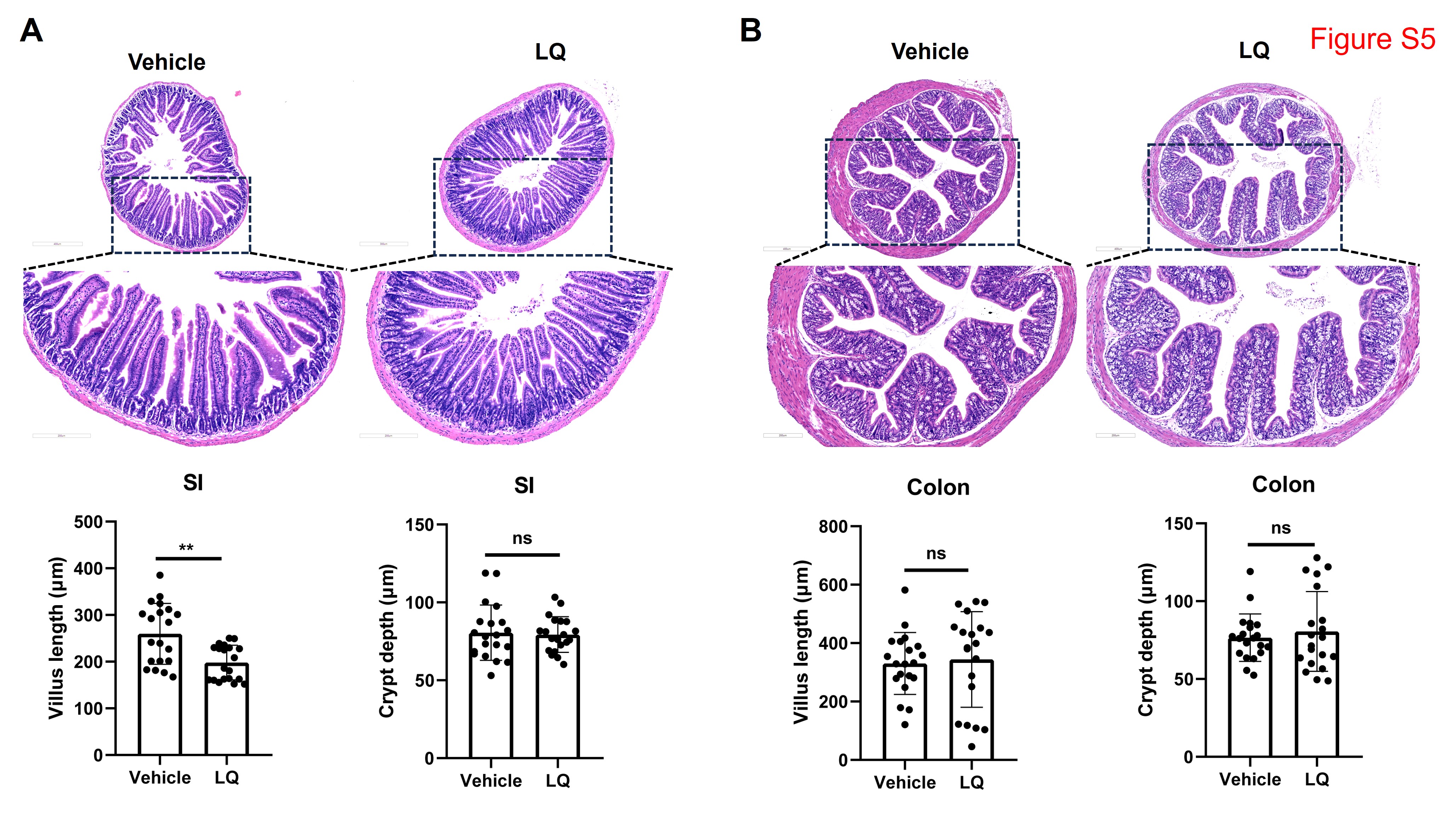

Supplement: Supplementary Figure S5 — Impact of Liquiritigenin Treatment on the Overall Intestinal Structure. (A, B) The overall intestinal structure of the small intestine (A) and colon (B) was assessed using hematoxylin and eosin (H&E) staining, and villus length and crypt length were quantified. [file Image5.jpeg]

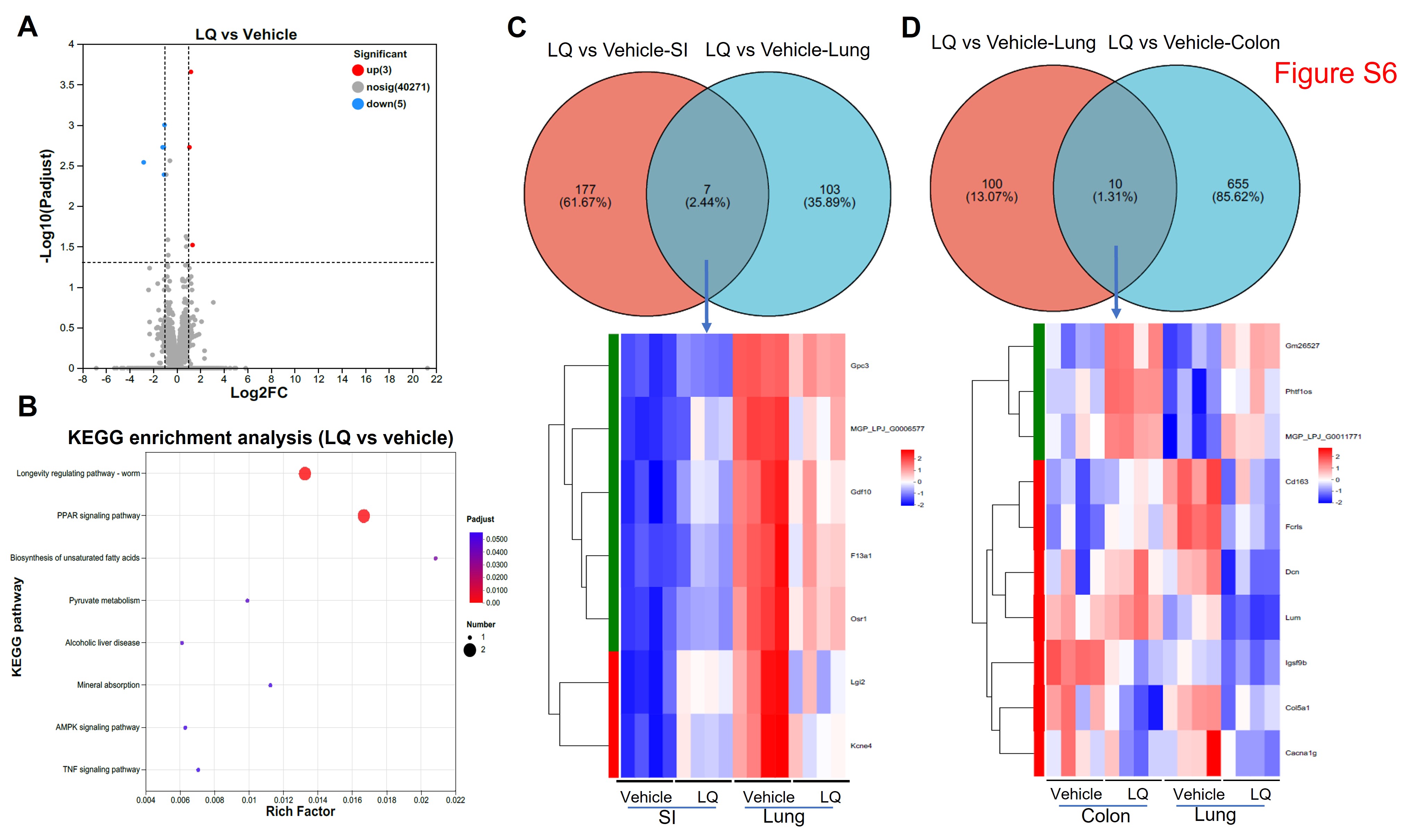

Supplement: Supplementary Figure S6 — Impact of Liquiritigenin Treatment on Gene Expression in Extraintestinal Tissues. (A) Volcano plots illustrating the differential expression of genes (DEGs) between the LQ and vehicle treated groups in the liver. (B) Kyoto Encyclopedia of Genes and Genomes (KEGG) enrichment analysis of DEGs in the LQ group compared to the vehicle group in the liver. (C) Heatmap illustrating the expression pattern of tissue-shared differentially expressed genes (DEGs) in the small intestine vs lung. (D) Heatmap illustrating the expression pattern of tissue-shared DEGs in the colon vs lung. [file Image6.jpeg]
